# Alantolactone reduced neuron injury via activating PI3K/Akt signaling pathway after subarachnoid hemorrhage in rats

**DOI:** 10.1371/journal.pone.0270410

**Published:** 2022-06-24

**Authors:** Feng Zhou, Zhenzhi Wang, Kang Xiong, Meiling Zhang, Yuan Wang, Maode Wang

**Affiliations:** 1 Department of Neurosurgery, First Affiliated Hospital of Xi’an Jiaotong University, Xi’an, Shaanxi, China; 2 Department of Neurosurgery, the Affiliated Hospital of Shaanxi University of Chinese Medicine, Xianyang, Shaanxi, China; 3 Department of Chinese and Western Medicine, the Shaanxi University of Chinese Medicine, Xianyang, Shaanxi, China; 4 Combination of Acupuncture and Medicine Innovation Research Center, Shaanxi University of Chinese Medicine, Xianyang, Shaanxi, China; Chinese Academy of Medical Sciences and Peking Union Medical College, CHINA

## Abstract

Subarachnoid hemorrhage (SAH) is a common disease with high morbidity and mortality, which can cause pathological, physiological, and biological reactions. SAH causes a series of responses such as neuronal and cerebral cortex damage, which in turn leads to inflammation and apoptosis. Traditional Chinese medicine has a strong anti-inflammatory effect, such as Alantolactone (ATL). However, studies on ATL therapy for SAH have not been reported. We observed the neurological scores, brain water content, Evans blue (EB) extravasation, neuroinflammation, and apoptosis via performing an enzyme-linked immunosorbent assay (ELISA), western blotting, immunofluorescence staining, and other methods after SAH. In this study, we found that ATL treatment attenuated the neurologic deficits, inhibited neuronal apoptosis and inflammatory reaction, promoted polarization of microglia toward the M2 phenotype, and activated the PI3K/Akt signaling pathway. ATL can reduce the neurons and cerebral cortex damage of SAH rats through activating PI3K/Akt signaling pathway.

## Introduction

Subarachnoid hemorrhage (SAH) is a common neurological disease, which could be life-threatening for people [1, 2]. As a fatal disease, SAH has been increasing in the world in recent years [3]. However, with the improvement of the medical environment and the continuous development of medical technology, the mortality rate of clinical patients has been greatly reduced [4, 5]. In the past 30 years, the mortality rate of SAH has decreased by about 17% [6]. However, various complications and functional defects encountered by the surviving patients in the recovery period are still important factors affecting their quality of life [7]. Although many SAH patients can achieve self-care after treatment, they show different degrees of anxiety, depression, and other mental symptoms, leading to their return to normal social life [8]. Thus, new treatment approaches are urgently needed.

Why is SAH such a serious disease? That is because arterial blood deposition makes severe neurological dysfunction and complications [9, 10]. For this kind of common disease with high incidence and threat to human health, the treatment strategies are also constantly developing and improving. However, many survivors after SAH have long-term cognitive impairment, and SAH brings huge economic and social burdens [11]. Besides, drug treatment options for SAH are still very narrow, although some studies on SAH physiopathologic mechanisms have been reported [12, 13]. Thus, a basic therapeutic strategy targeting SAH is still important.

Traditionally, delayed cerebral vasospasm is considered to be the main cause of the poor prognosis of SAH. However, in clinical trials, vasospasm, after SAH and secondary delayed ischemic neurological deficit were, was not obvious [14, 15]. Therefore, early brain injury (EBI) is defined as a poor prognosis within 48 h after SAH [16, 17]. SAH induces neuronal and white matter injury which increased intracranial pressure, neuroinflammation, brain edema, and blood-brain barrier (BBB) damage [12]. Recent studies reported that SAH triggered the release of pro-inflammatory cytokines, such as including IL-1β, nitric oxide, and tumor necrosis factor-α, which was a key mediator of neuronal apoptosis after SAH [18, 19]. In addition, SAH were found to induce neuronal apoptosis via reducing BCL-2 and increasing Bax in microglia [20]. While some signaling pathways have been associated with anti-apoptosis and anti-inflammatory, and brain injury, such as TLR4/ NF-κB [21], NLRP3 inflammasome [22], and PI3K/Akt signaling pathway [12, 23]. The current research approach is to investigate the pathological changes at 48 h after SAH, and there is an urgent need for potential new drugs with comprehensive therapeutic effects.

Traditional Chinese medicine has a long history and plays an important pharmacological role. In recent years, traditional Chinese medicine has been paid to the attracted. In the anti-bacterial and anti-tumor activities, Alantolactone (ATL) has been concerned widely [24]. ATL is a sesquiterpene lactone compound, which was recorded in the medical book *Bencao Tujing* [25]. Besides, ATL could play neuroprotective roles after a rat’s brain is injured [24]. ATL could also penetrate BBB to perform the inhibitory effect [26]. However, ATL has not been well used for the SAH treatment strategy. In all, it is a hypothesis that ATL has an effective pharmacological effect for SAH treatment.

## Material and methods

### Animals and SAH model

A total of 120 Healthy male Sprague-Dawley (SD) rats weighed at 320–350 g were purchased from the Animal Center of Xi’an Jiaotong University. All experiments were performed following the National Institutes of Health Guide for the Care and Use of Laboratory Animals (NIH Publications No. 8023, revised 1978) and were approved by the Animal Care Committee of the Affiliated Hospital of Shaanxi University of Chinese Medicine, and the permit numbers were SUCMDL20180313015. Animals were sacrificed by using an overdose of sodium pentobarbital, and all efforts were made to minimize suffering.

The rats were used as the control group (sham) and SAH group. SAH model was conducted by the improved method of intravascular puncture [27]. Briefly, rats were anesthetized with by inhalation of 5% isoflurane initially. After intubation and initiation of mechanical ventilation, anesthesia was maintained with 2.5 to 3% isoflurane and supine fixation. The rectal temperature was controlled at 36±1˚C with a heating pad. Then, the neck median incision was taken, and the carotid artery, external carotid artery, and internal carotid artery were exposed. 4–0 nylon thread was inserted through the external carotid artery and pushed forward to the neck. The nylon thread was pulled out 10 seconds after a successful puncture, the external carotid artery was ligated, the aneurysm clip was removed, and the internal carotid artery blood flow was restored. Finally, the wound was sutured layer by layer and disinfected. The sham group underwent the same procedure, while nylon thread did not puncture the blood vessel after insertion. After completing the procedures, a subcutaneous injection of 20 ml 0.9% NaCl solution was given and the rats were placed in a 24 ± 1°C incubator until they recovered. The veterinarians guided the animal care including daily observation (every 12 hours per day) and neurological scoring. Rats were checked frequently, approximately every 10–15 minutes, and turned side to side until they recovered. The dead rats which were calculated in the mortality included those which died on their own beyond the observation and those which were euthanized because of bad conditions. The following conditions are the criteria for euthanasia: complete anorexia for 24 h; inability to obtain feed or water; infection (non-healing wounds, organ infection); dyspnea.

### Experimental grouping

120 rats were randomly divided into the following six groups: (1) Sham group; (2) ATL(M) group (SD rats were intraperitoneally injected of ATL with 20 mg/kg); (3) SAH group (SAH rats); (4) SAH+ATL(L) group (SAH rats were intraperitoneally injected of ATL with 10 mg/kg); (5) SAH+ATL(M) group (SAH rats were intraperitoneal of ATL with 20 mg/kg); (6) SAH+ATL(H) group (SAH rats were intraperitoneally injected of ATL with 40 mg/kg). Rats were fed with a general diet and free access to water in the animal house with 12 h: 12 h (light: dark). Then, the sham rats and SAH rats were operated. At 30 min after the operation, each group was received ATL by an *i*.*p*. injection, and ATL (purity > 98%) was purchased from CHENGDU MUST BIO-TECHNOLOGY CO., LTD (cat no. A0221), and another dose was administered 24 h later, twice in total. After 48 h with the SAH modeling, the animals were sacrificed by cervical dislocation after being anesthetized by an intraperitoneal injection of 2% pentobarbital sodium (35 mg/kg).

### Evaluation of SAH severity

The severity of SAH was blindly evaluated using high-resolution pictures of the base of the brain taken at each sacrifice [28]. In brief, the basal brain including brainstem was divided into 6 segments, and each segment was allotted a grade from 0 to 3 depending on the amount of subarachnoid blood clot. The minimum score is 0 and the maximum is 18.

### Neurological evaluation

After 48 h with the SAH modeling and different doses of ATL injection, the neurological evaluation was assessed. Neurological evaluation consisted of the improved Garcia score and beam balance test [29, 30]. The average score of three consecutive trials has been calculated. The higher score, the better test performance got. The observers were blind to all group information.

### Brain water content

Wet-dry weight method was used to test the brain water content [31]. The brain was sampled after treatment. Then, the brain was weighed after removing the meninges and blood clots on the surface, which was wet weight. The wet brain was dried for 48 h at 100°C in an oven and reweighed as dry weight. After removing the meninges and blood clots on the surface, the brain tissue mass was immediately weighed to as the wet weight. The percentage of brain water content was calculated as [(wet weight—dry weight)/ wet weight] ×100%.

### Evans blue (EB) extravasation assay

BBB disruption was determined by quantifying the EB leakage into brain tissue [32]. Briefly, rats were injected intravenously with EB at a dose of 100 mg/kg, which stably binds with albumin in the blood. After 5 min, rats were injected with BLs (100 μg/rat) and the right hemispheres of rats were exposed with a 3.5 MHz pulsed HIFU (10% duty, 10-60s) with different intensities (0.5–1.5 kW/cm^2^). After several hours, the treated mice were infused intravenously with PBS as a perfusion medium using a syringe pump at a constant speed. The mice were perfused with PBS via the left ventricle. After the perfusion and brain removal, the brains were then divided into the right and left hemispheres before measuring the amount of EB that was extravagated. The non-exposed left hemispheres of the treated mice were used as the control. The samples were weighed, soaked in formamide solution, and incubated for 24h at 55°C. Subsequently, the extracted dye concentration was determined using a spectrophotometer at 620nm. To understand the effect of BLs with HIFU exposure on the duration of BBB permeability at the focused site, the mice were intravenously injected with BLs, and the right hemispheres were exposed to HIFU. After 5 min, 30 min, 3 h, or 24 h, the mice were intravenously injected with EB. The amount of EB extravasation in each brain was examined after 3 h after the EB injection, as described above.

### Western blot

Rats in each group were euthanized by saline perfusion in the left ventricle until the right atrium was unobligated. Total proteins were extracted from brain tissue from the left hemispheres with RIPA lysate. Following estimated the protein concentration using a bicinchoninic acid (BCA) kit, 50 μg total proteins were separated by 10% SDS-PAGE gel and then transferred onto PVDF membranes (Millipore, MA, USA). After being blocked in Tris-buffered saline with Tween-20 (TBST) within 5% milk for 1 h, PVDF membranes were incubated at 4°C overnight with the primary antibodies against PI3K, Akt, p-Akt. All antibodies were purchased from Abcam (Cambridge, MA, UK). Then, the membranes were washed with TBST and incubated with HRP-conjugated goat anti-rabbit immunoglobulin G secondary antibody (BA1054; Wuhan Boster Biological Technology, Ltd.) at a dilution of 1:5,000 for 1 h at room temperature. Enhanced chemiluminescence reagent (Millipore) was used to detect the signals on the membranes using the Bio-Rad ChemiDocTMMP system (Bio-Rad Laboratories, Inc., Hercules, CA, USA). Image-ProPlus software (Media Cybernetics, Inc., Rockville, MD, USA) was used to quantify bands, and β-actin was used as a loading control.

### Enzyme-linked immunosorbent assay (ELISA)

The protein homogenates of the rat’s brain left hemisphere were used for the detection of a cytokine through ELISA. Immune-mediators tumor necrosis factor-a (TNF-a), Interleukin-1β (IL-1β), IL-6, and IL-10 were measured by ELISA kits according to the manufacturer’s instructions (Nanjing Jiancheng Bioengineering Institute, Jiangsu, China).

### H&E, TUNEL, and Nissl staining

The ipsilateral temporal cortex was taken for H&E, TUNEL, and Nissl staining. Briefly, the brain tissues were fixed with 4% paraformaldehyde over 24 h, embedded in paraffin blocks, and then prepared for 4-μm-thick paraffin sections. For H&E staining, slices were stained with hematoxylin and eosin, and the pathologic change was observed using an optical microscope. TUNEL staining was used to label apoptotic cells by fluorescent-TdT enzyme in a FragEL™ DNA Fragmentation Detection Kit (Calbiochem, Darmstadt, Germany). The apoptotic cells exhibited brown staining within the nucleus. Images were captured with fluorescence microscopy (Olympus, IX71, Olympus Co., Tokyo, Japan). For Nissl staining, the sections were fixed on the polylysine-coated slides, dried overnight, rehydrated in distilled water, and then immersed in 1% cresyl violet for 20min. After being rinsed with distilled water and dehydrated by graded serried of ethanol, these sections were submerged in xylene and then coverslipped. Nissl-positive cells in the ipsilateral temporal cortex were observed to assess neuronal loss under a microscope (Leica, DM6000 B, Tokyo, Japan). Round neurons with pale nuclei are considered to be viable cells, and condensed and shrunken neurons are considered as damaged cells. The average number of Nissl-positive cells in six random visual fields per section in penumbra was used for statistical analysis.

### Immunofluorescence assay

After air-dried for 30 min at room temperature, sections were rinsed with 0.1 M PBS to remove the OCT compound. And then sections were treated with blocking buffer (PBS, 2% BSA (Sigma Aldrich, America), and 0.2% Triton X-100) followed by incubation with goat anti-Iba1, anti- CD16, or anti-CD206 overnight at 4°C. Following the rinse 3x10 min with 0.1 M PBS, sections were incubated with corresponding secondary antibodies for 1 h at room temperature. Sections were mounted using Mounting Medium, antifading (with DAPI) (S2110, Solarbio) after being washed 3 times in 0.1 M PBS. Sections were observed by fluorescence microscopy (Olympus, IX71, Olympus Co., Tokyo, Japan).

### Statistical analysis

All statistical values were calculated using SPSS 20 software analysis the statistical values. Data were represented as means ± standard deviation (SD). Normality was assessed using the Shapiro-Wilk normality test. Homogeneity of variances was checked using the Levene test. One-way analysis of variance (ANOVA) and Tukey’s-b post hoc test were used to compare differences among multiple groups. The differences were considered statistically significant at p < 0.05 and p < 0.01.

## Results

### Mortality and SAH grade

**Fig 1:**
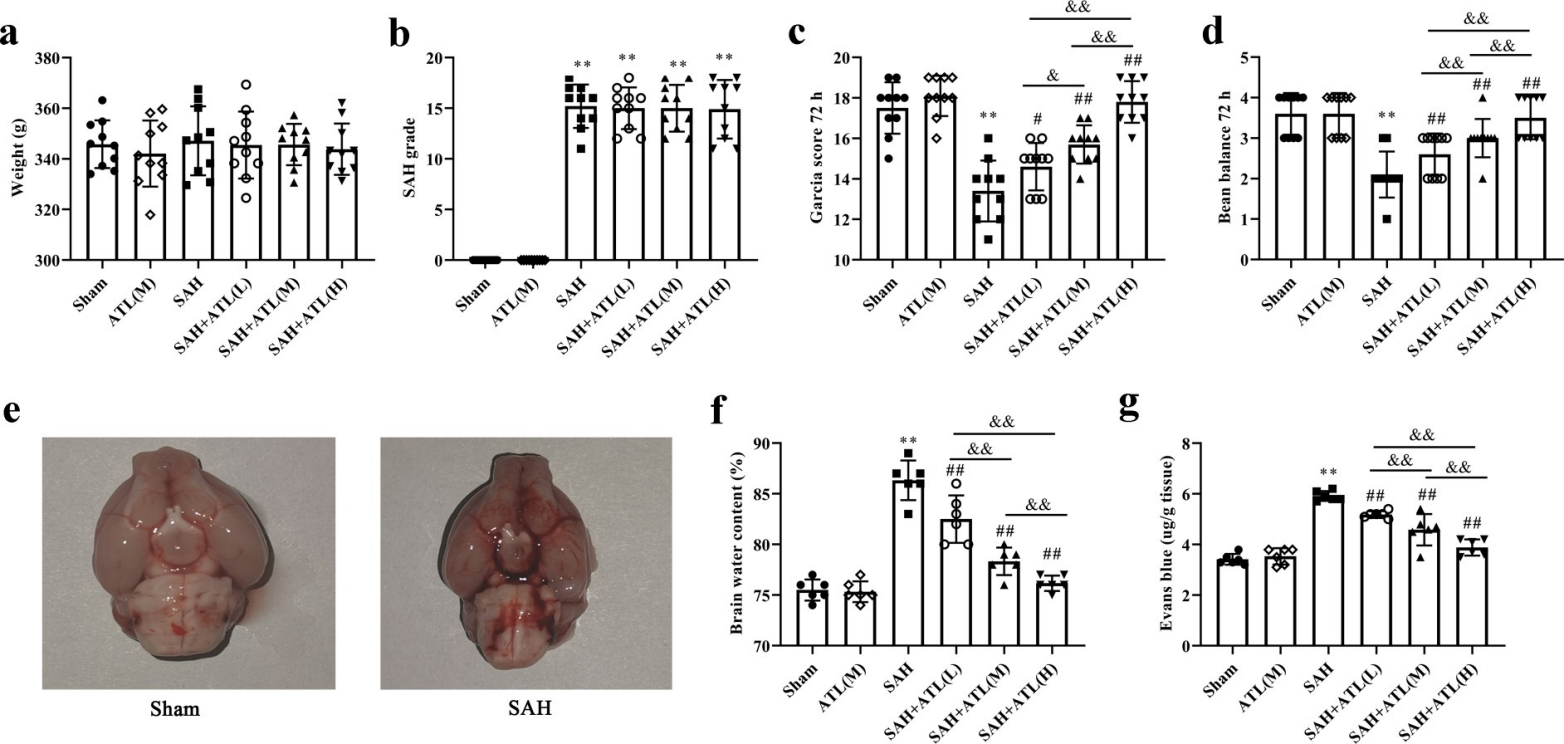
Effects of ATL on neurologic deficits, brain edema and BBB disruption at 72h after SAH. The Weight (a), SAH grade (b), Garcia score (c), beam balance (d), the whole brain (e), brain water content (f), EB extravasation (g) among each group. Data are represented as the mean ± SD. **p<0.01 vs. sham group; ^#^*p*<0.05 and ^##^*p*<0.01 vs. SAH group; ^&^*p*<0.05 and ^&&^*p*<0.05. a-d, n = 10, the number of animals; f and g, n = 6, the number of animals.

Bodyweight among the groups did not have obvious change (Fig 1A). There was no significant difference in the mean SAH grade between all SAH groups (Fig 1B). No rats died in the Sham group (0/12 rats) and ATL(M) group (0/12 rats). The mortality rates were 28%, 27.8%, 17.9% and 10.7% in SAH group (7/25 rats), SAH+ATL(L) group (5/18 rats), SAH+ATL(M) group (5/28 rats), SAH+ATL(H) group (3/28 rats), respectively. Three rats were excluded from this study due to the low grade (< 8) SAH at 24 h.

### ATL attenuated neurologic deficits, brain edema, and BBB disruption at 48 h after SAH

The Garcia score and beam balance score of the SAH group were significantly decreased compared with the sham group (*P*<0.01), while the scores of the ATL group were in a dose-dependent increase (*p*<0.05, Fig 1C and 1D). There was a significant hemorrhage in brain tissue of the SAH group (Fig 1E). Compared with the sham group, the water content and EB exudation were significantly increased in the SAH group (*p* < 0.01), while the water content and EB exudation decreased in a dose-dependent manner in the ATL group (*p*<0.01, Fig 1F and 1G).

### ATL attenuated brain injury at 48 h after SAH

**Fig 2:**
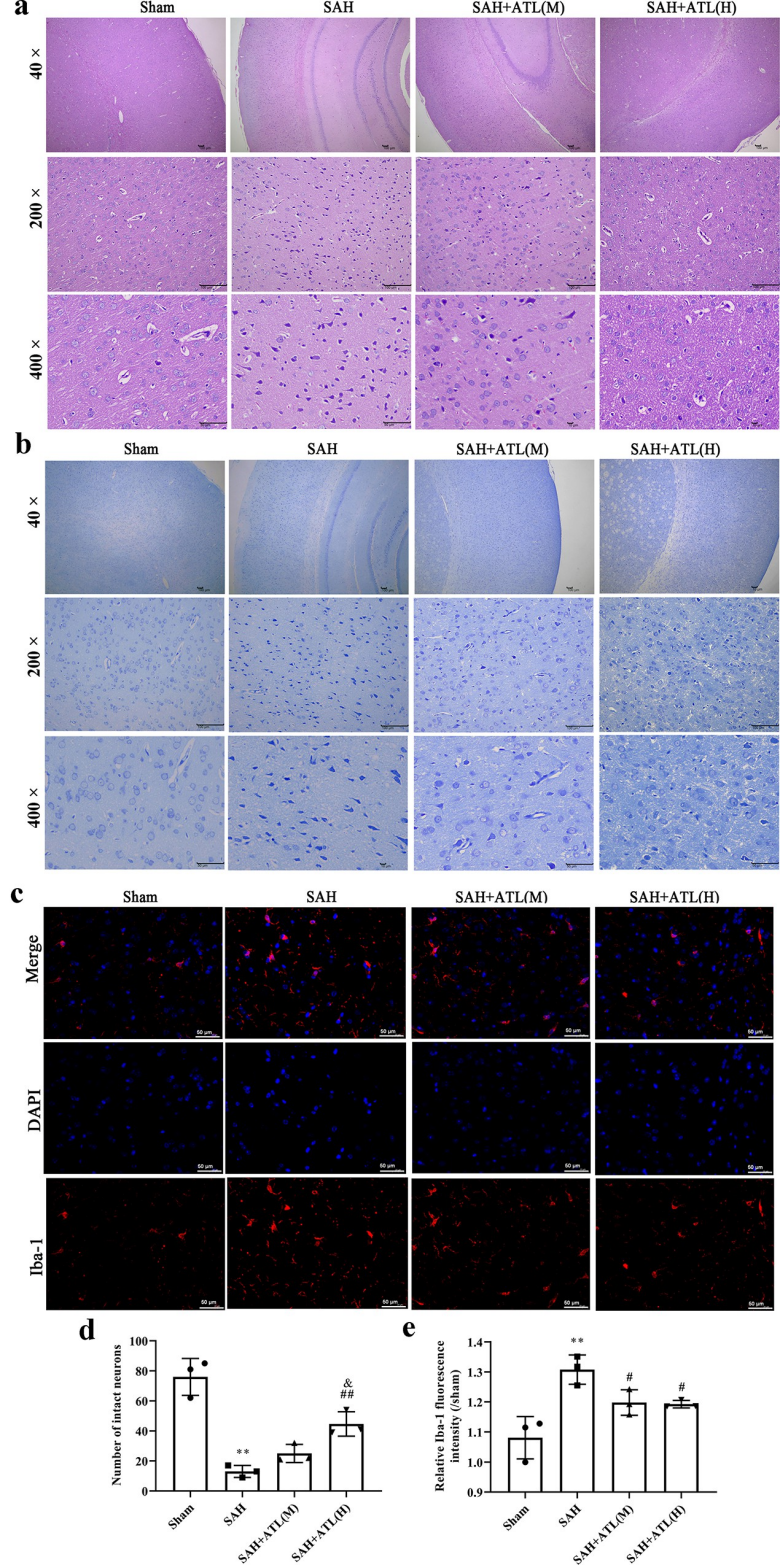
Effects of ATL on the morphological changes of neurons at 72h after SAH. (a) Representative images of H&E staining of the ipsilateral temporal cortex in different groups. Up means 20 x magnifications (Scale bar 100 μm), down means 40 x magnification (Scale bar 50 μm). (b, d) Representative images of Nissl staining of the ipsilateral temporal cortex (b) and quantitative analysis of the number of intact neurons (d). Scale bar 50 μm. (c, e) Representative images of immunofluorescence staining (c) and quantitative analysis (e) of the Iba-1 of the ipsilateral temporal cortex. Scale bar 20 μm. Data are represented as the mean ± SD. **p<0.01 vs. sham group; ^#^*p*<0.05 and ^##^*p*<0.01 vs. SAH group; ^&^*p*<0.05 vs. SAH+ATL group. n = 3, the number of images analyzed.

Morphological changes in the ipsilateral temporal cortex were measured with H&E staining (Fig 2A). Compared with the sham and SAH+ATL(H) group, a large number of neurons were necrotic, cytolysis, nucleus pyknosis, and hyperplasia of microglia in the SAH group. Nissl staining showed that intact neuronal cells were exhibited in the sham group, while lots of damaged neurons were exhibited in the SAH group (*p*<0.01, Fig 2B and 2D). However, ATL treatment could attenuate the neuronal injury (*p*<0.01, Fig 2B and 2D). Inflammatory stimuli induce microglia to morph from a ramified to an amoeboid shape. As shown in Fig 2C and 2E, immunofluorescence results showed that the number of activated microglial cells in the ipsilateral temporal cortex was significantly increased after SAH compared with the sham group (*p*<0.01), while ATL can remarkably reduce the number of activated microglial cells (*p*<0.05).

### ATL attenuated neuronal apoptosis at 48 h after SAH

**Fig 3:**
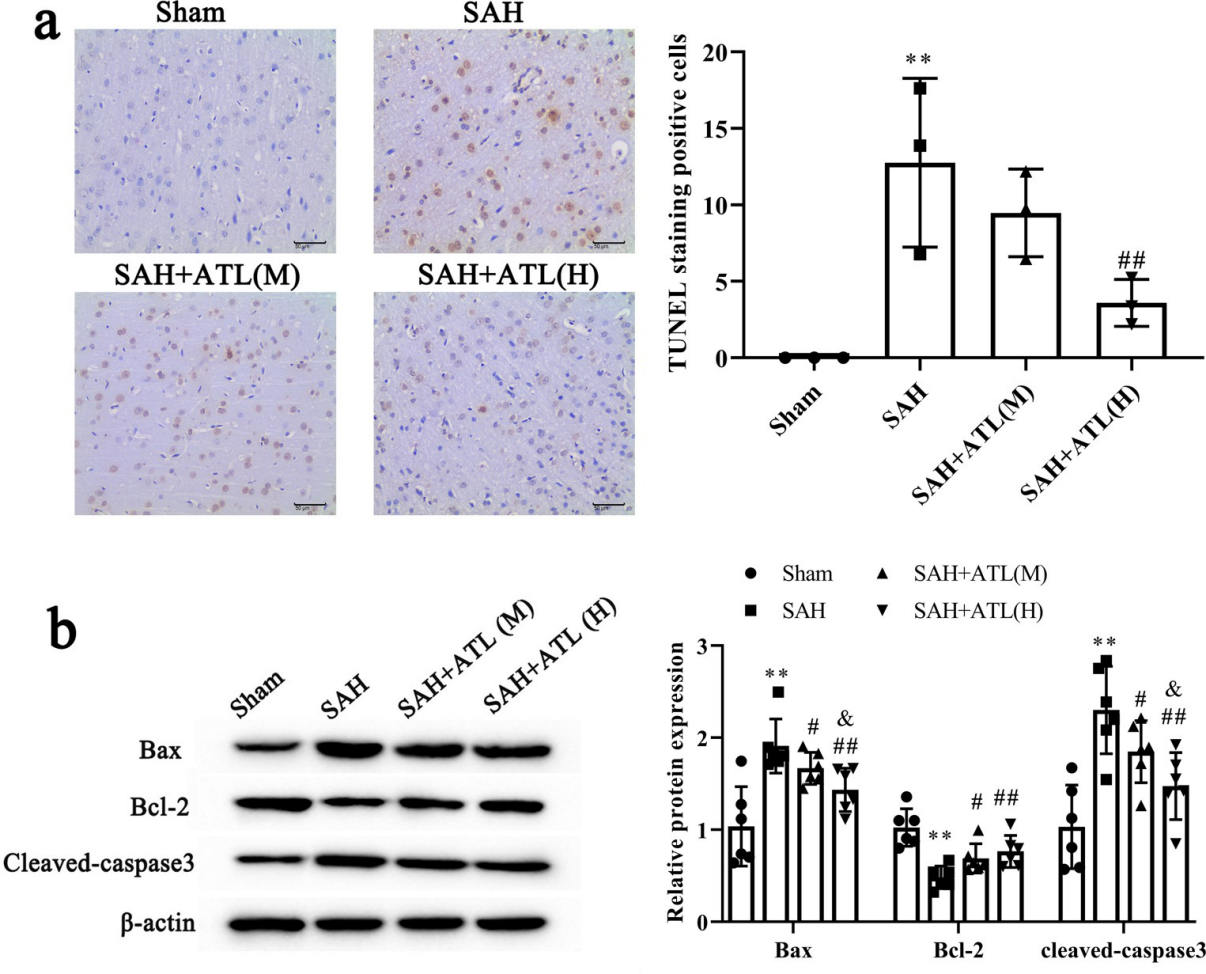
Effects of ATL on neuronal apoptosis at 72h after SAH. (a) Representative images of TUNEL staining of the ipsilateral temporal cortex at 72 h after SAH and quantitative analysis. Scale bar = 50 μm. n = 3, the number of images analyzed. (b) protein expression of Bax, Bcl-2, and cleaved-Caspase 3 among each group. n = 6. Data are represented as the mean ± SD. **p<0.01 vs. sham group; ^#^*p*<0.05 and ^##^*p*<0.01 vs. SAH group; ^&^*p*<0.05 and ^&&^*p*<0.05.

Abundant TUNEL positive cells were detected in the SAH group compared with the sham group, while the high dose ATL injection reduced TUNEL-positive cells significantly (*p*<0.01, Fig 3A). Furthermore, western blot results showed that the apoptotic marker protein expression including Bax and cleaved-Caspase 3 was significantly increased at 48 h after SAH compared with the sham group (*p*<0.01), while the high dose ATL injection reduced Bax and cleaved-Caspase 3 expressions (*p*<0.01, Fig 3B). The protein expression of Bcl-2 was significantly decreased at 48 h after SAH compared with the sham group (*p*<0.01), while ATL injection increased Bcl-2 expression (*p*<0.05, Fig 3B).

### ATL attenuated inflammatory reaction at 48 h after SAH

**Fig 4:**
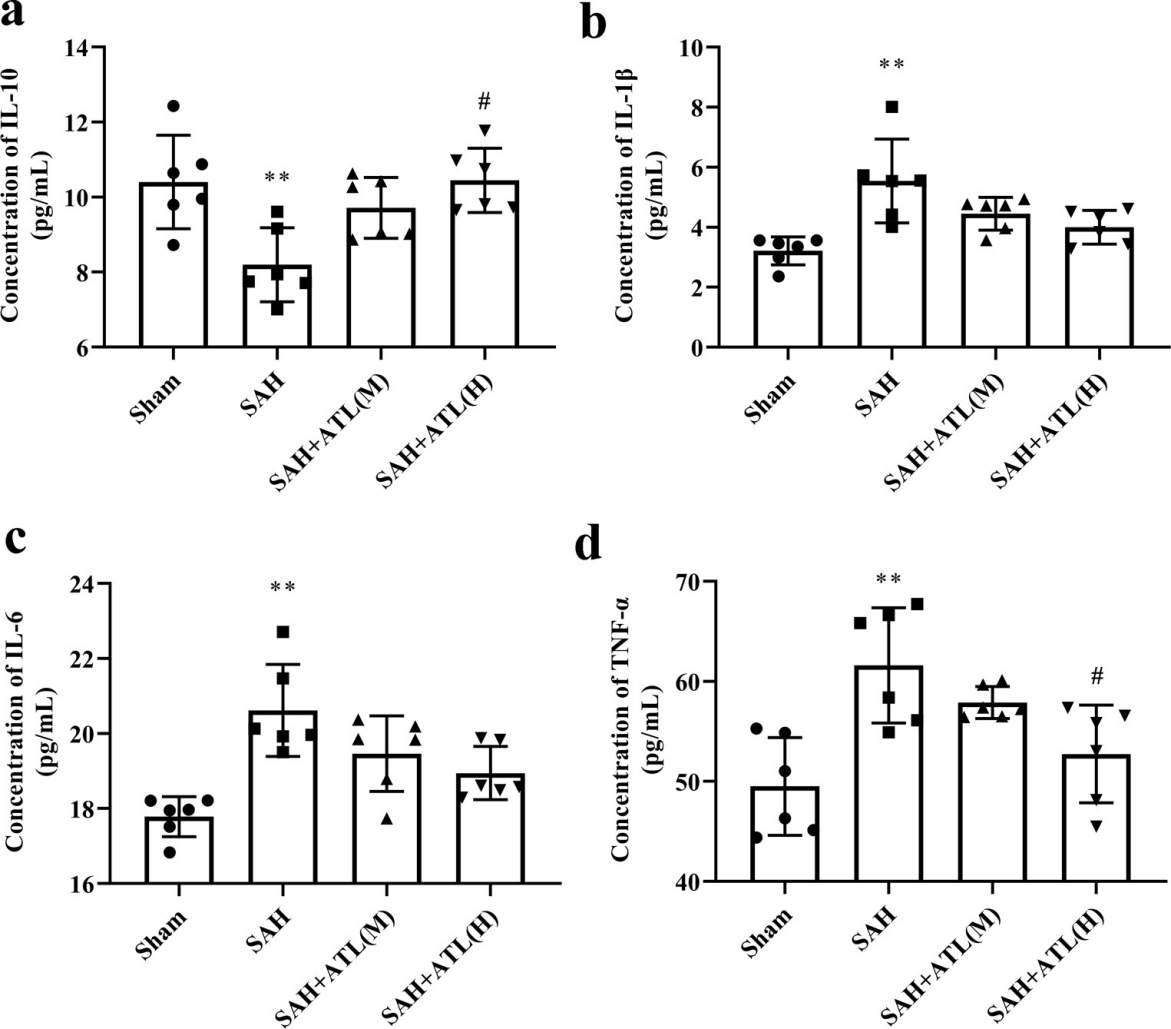
Effects of ATL on inflammatory reaction at 72h after SAH. The concentration of IL-10 (a), IL-1β (b), IL-6 (c), and TNF-α (d) of the ipsilateral temporal cortex at 72h after SAH. Data are represented as the mean ± SD. *p<0.05 and **p<0.01 vs. sham group; ^#^*p*<0.05 vs. SAH group. n = 6, the number of animals.

**Fig 5:**
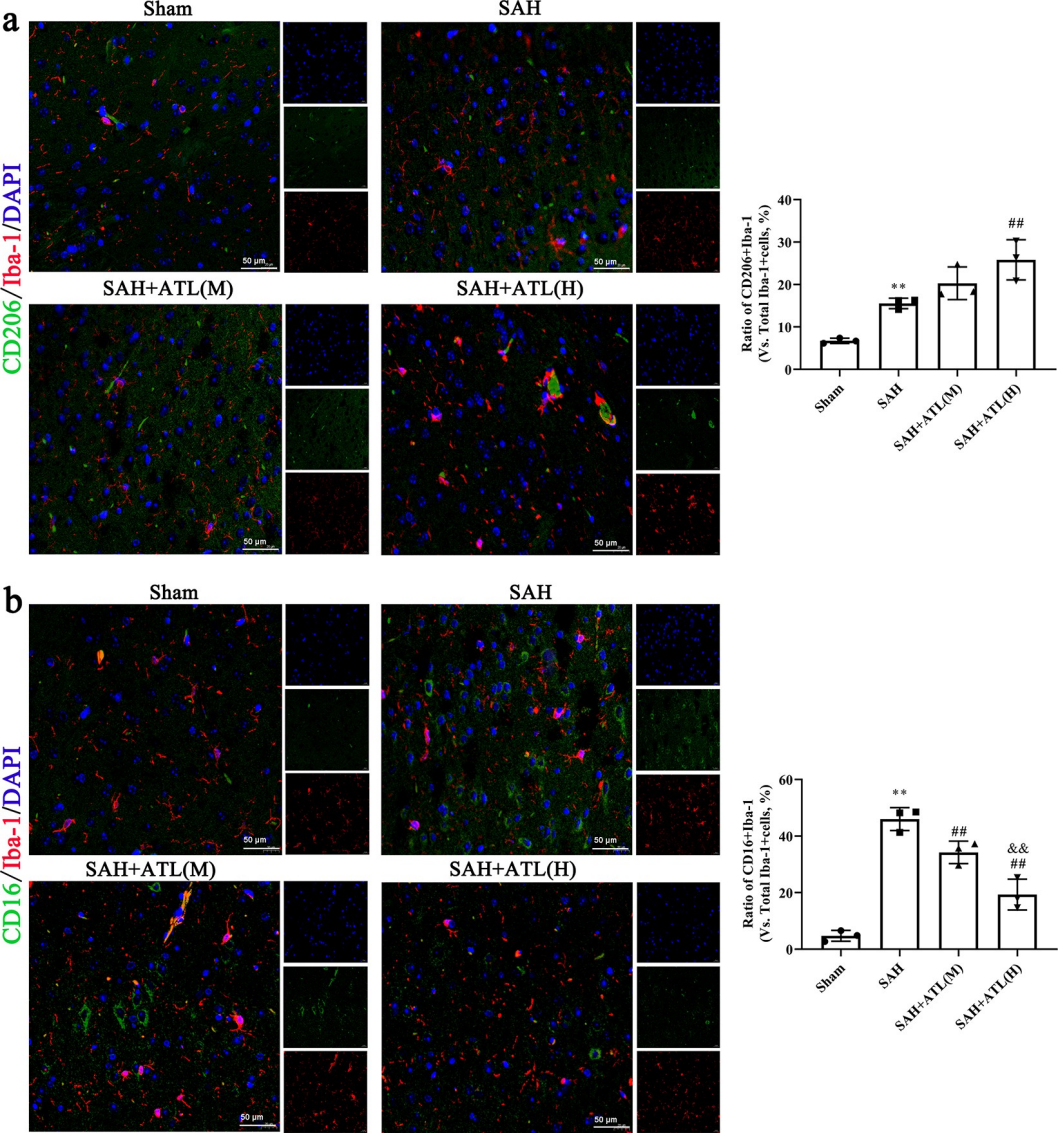
Effects of ATL on microglia polarization at 72h after SAH. (a) immunofluorescent staining against CD206/Iba-1 of the ipsilateral temporal cortex at 72h after SAH. Scale bar = 20 μm. (b) immunofluorescent staining against CD16/Iba-1of the ipsilateral temporal cortex at 72h after SAH. Scale bar = 20 μm. Data are represented as the mean ± SD. **p<0.01 vs. sham group; ^##^*p*<0.01 vs. SAH group; ^&&^*p*<0.05. n = 3, the number of images analyzed.

Microglia can be recruited into brain tissue through the expression of chemokines and cell adhesion molecules, leading to the release of cytokines such as IL-1β and TNF-α, thus aggravating the inflammatory response. In the present study, the concentration of IL-1β, IL-6, and TNF-α showed a marked increasing level at 48 h after SAH compared with the sham group (*p*<0.01), while ATL slightly reduced the levels of IL-1β and IL-6, although the results were not statistically significant (*p*>0.05, Fig 4B and 4C). As expected, ATL high dose significantly decreased the content of TNF-α (*p*<0.05). Besides, the concentration of IL-10 was decreased significantly at 48 h after SAH (*p*<0.01), while significantly increased by ATL high dose (p<0.05, Fig 4A) and softly enhanced by ATL low dose injection (*p*>0.05, Fig 4A). Subsequently, the numbers of CD16+/Iba‐1+ M1‐type microglia and CD206+/Iba‐1+ M2‐type microglia microglia were counted. Immunofluorescence results showed the number of CD16 and CD206 positive Iba-1 cells after SAH at 48 h was both significantly increased compared with the sham group (p<0.01, Fig 5A and 5B). Following ATL admission, the number of CD16+/Iba‐1+ was significantly decreased (*p*<0.01), while the number of CD206+/Iba‐1+ cells was significantly increased (*p*<0.01).

### ATL activated PI3K/Akt signaling pathway after SAH

**Fig 6:**
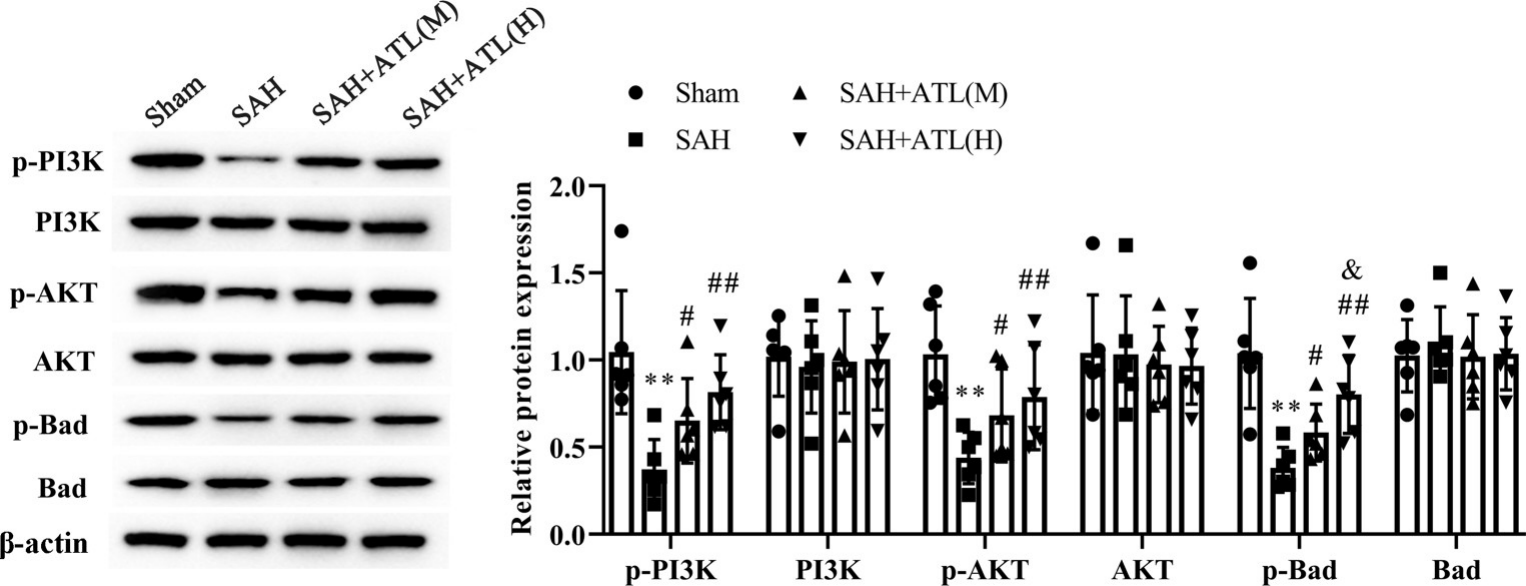
Effects of ATL on the PI3K/Akt signaling pathway at 72h after SAH. Protein expression of p-PI3K, PI3K, Akt, p-Akt, p-Bad, and Bad among each group of the ipsilateral temporal cortex at 72 h after SAH. Data are represented as the mean ± SD. **p<0.01 vs. sham group; ^#^*p*<0.05 and ^##^*p*<0.01 vs. SAH group. n = 6, the number of animals.

The western blot result showed that there is no significant obvious on the protein expressions of PI3K, Akt, and Bad among each group (*p*>0.05, Fig 6). However, the protein expression of p-PI3K, p-Akt, and p-Bad decreased significantly at 48 h after SAH (*p*<0.01) and increased after ATL treatment (*p*<0.05, Fig 6).

## Discussion

Brain injury after SAH occurs in a series of the pathological process, and the inflammatory response and cell apoptosis are crucial mechanisms that mediate the subsequent histopathology and neurobehavioral deficits. In our study, a rat model was used to evaluate the protective effects of ATL after SAH, and the potential molecular mechanisms and results are as followed: (a) SAH induced significant inflammation, cerebral cortex injury, brain edema, and cell apoptosis; (b) after ATL treatment, early brain damages were ameliorated after SAH; and (c) ATL could play neuroprotective roles through activating PI3K/Akt signaling pathway. In all, these findings indicate that ATL could attenuate the neuron and cerebral cortex injury through activating PI3K/Akt signaling pathway in SAH rats.

In the SAH model, the one-hemorrhage or two-hemorrhage SAH model was used in previous studies. However, the injection-hemorrhage model is not suitable for the study of early brain injury in SAH. Therefore, compared with the injection hemorrhage model, the intravascular puncture model may be more suitable for the study of early brain injury in SAH [33]. In our study, SAH induced a series of pathological reactions, including neurologic deficits, brain edema, BBB disruption, cell apoptosis, and inflammatory reaction. The results show that ATL treatment has a remarkable recovery effect. The medicinal plant is mild in nature, bitter in taste, non-toxic, and healthy in the lung, liver, and spleen channels. It is commonly used to treat chest and flank pain, vomiting and diarrhea, dysentery, and so on. ATL, a sesquiterpene lactone with the molecular formula of C_15_H_20_0, is one of the main active components of daphne odora [24]. In recent years, studies have shown that ATL has many pharmacological activities, such as antitumor [34, 35], antibacterial [36], anti-inflammatory [37, 38], liver-protecting [39, 40], and so on. However, the relationship between ATL and SAH has not been reported. To the best of our knowledge, the present study is the first to found identify that ATL may be therapeutic after SAH.

In the present, ATL could ameliorate EBI in SAH by reducing neuroinflammation and neuronal apoptosis. Microglia are important immune cells in the central nervous system (CNS). Under pathological conditions such as ischemia/hypoxia, trauma, and toxic substances stimulation, microglia were activated and released a large number of pro-inflammatory factors, chemokines, reactive oxygen species (ROS), and other mediators, thereby aggravating brain tissue injury [41, 42]. Activated microglia have two opposed polarization types: one called classically activated M1-type microglia (from a resting state to pro-inflammatory effects), and the other called selectively activated M2-type microglia (anti-inflammatory responses) [43, 44]. It has been shown that SAH induced microglia activation, and promoted the level of M1-type microglial markers including IL-1β, iNOS, IL-6, TNF-α, MCP-1, and NLRP3 inflammasome [45]. Furthermore, SAH caused the microglia polarization shift from the M2 phenotype and skewed toward the M1 phenotype [46], evidenced by the increased levels of IL-6, IL-1β, and TNF-α [47]. Similarly, our data showed that SAH promoted the expression of pro-inflammatory, suppressed IL-10 expression, and stimulated changes in polarization from the M2 to the M1 phenotype in microglia, which were reversed by ATL treatment. Microglial activation was considered harmful for neurons and caused neuronal apoptosis. Literature reports suggested that the anti-apoptotic proteins Bcl-2, Bcl-XL were decreased, and the apoptotic protein cleaved caspase 3, Bax were increased after SAH injury. This study indicates that administration of ATL diminished SAH- induced neuronal apoptosis, which was characterized by the decreased expression of Bax and cleaved-Caspase 3, as well as increased expression of Bcl-2.

Our study has shown that ATL attenuated the neuron and white matter injury through PI3K/Akt signaling pathway. PI3K/Akt signaling pathway is a classic signal pathway regulating apoptosis. PI3K is a kind of intracellular phosphatidylinositol kinase [48]. Akt can phosphorylate a series of protein components in cells [49]. Bad is an important downstream target molecule of Akt, which belongs to the Bcl-2 anti-apoptosis-related gene [50]. Akt can directly phosphorylate bad to form monomers of Bcl-2, which plays an anti-apoptotic role [51]. It was found that PI3K/Akt signaling pathway played an important role in neuronal apoptosis and inflammatory reaction in SAH-induced brain injury [52, 53]. In SAH rats, fibroblast growth factor (FGF)-2 inhibited post-SAH neuronal apoptosis via activating the FGFR3/PI3k/Akt signaling pathway [54]. Aggf1 treatment attenuated neuroinflammation of SAH rats through the PI3K/Akt/NF-κB signaling pathway. We found that the expression levels of p-PI3K, p-Akt, and p-Bad in the SAH group were dramatically decreased, which was corroborated these previous reports. We also found that ATL promoted the phosphorylation levels of PI3K, Akt, and Bad in brain tissues of SAH rats. The previous report demonstrated that the cytochrome c/caspase-dependent apoptotic pathway partially mediated the ATL neuroprotective effect of ALT in a traumatic brain injury (TBI) rat model [52]. Furthermore, ALT inhibited neuroinflammation by inhibiting the NF-κB pathway and mitogen-activated protein kinase (MAPK) signaling in LPS-activated microglial cells [55]. Notably, our finding elucidates another pharmacological activity of ATL in regulating neuronal apoptosis and neuroinflammation.

In conclusion, this is the first study to demonstrate the role of ATL in the SAH model and demonstrate the anti-apoptosis and anti-inflammatory properties of ATL after SAH. Furthermore, ATL attenuated brain injury, neuronal apoptosis, and inflammatory reaction via activating PI3K/Akt signaling pathway after SAH. Thus, ATL may be a promising therapeutic option for patients with SAH in an early stage. Further studies including clinical trials are still needed to elucidate the potential of ATL as a neuroprotective agent for SAH.

## Supporting information

S1 Raw images (PDF)

## Abbreviations

SAH: Subarachnoid hemorrhage
ATL: Alantolactone
ELISA: enzyme-linked immunosorbent assay
EBI: early brain injury
BBB: blood-brain barrier
SD: Sprague-Dawley
BCA: bicinchoninic acid
PVDF: polyvinylidene difluoride
TNF-a: tumor necrosis factor-a
IL-1β: Interleukin-1β
SD: standard deviation
ANOVA: One-way analysis of variance
