# Supplementary figures and images for: Alantolactone reduced neuron injury via activating PI3K/Akt signaling pathway after subarachnoid hemorrhage in rats
Source: PLoS One. 2022 Jun 24;17(6):e0270410. doi: 10.1371/journal.pone.0270410 (PMC9231788; doi:10.1371/journal.pone.0270410)

Bax (n=6)

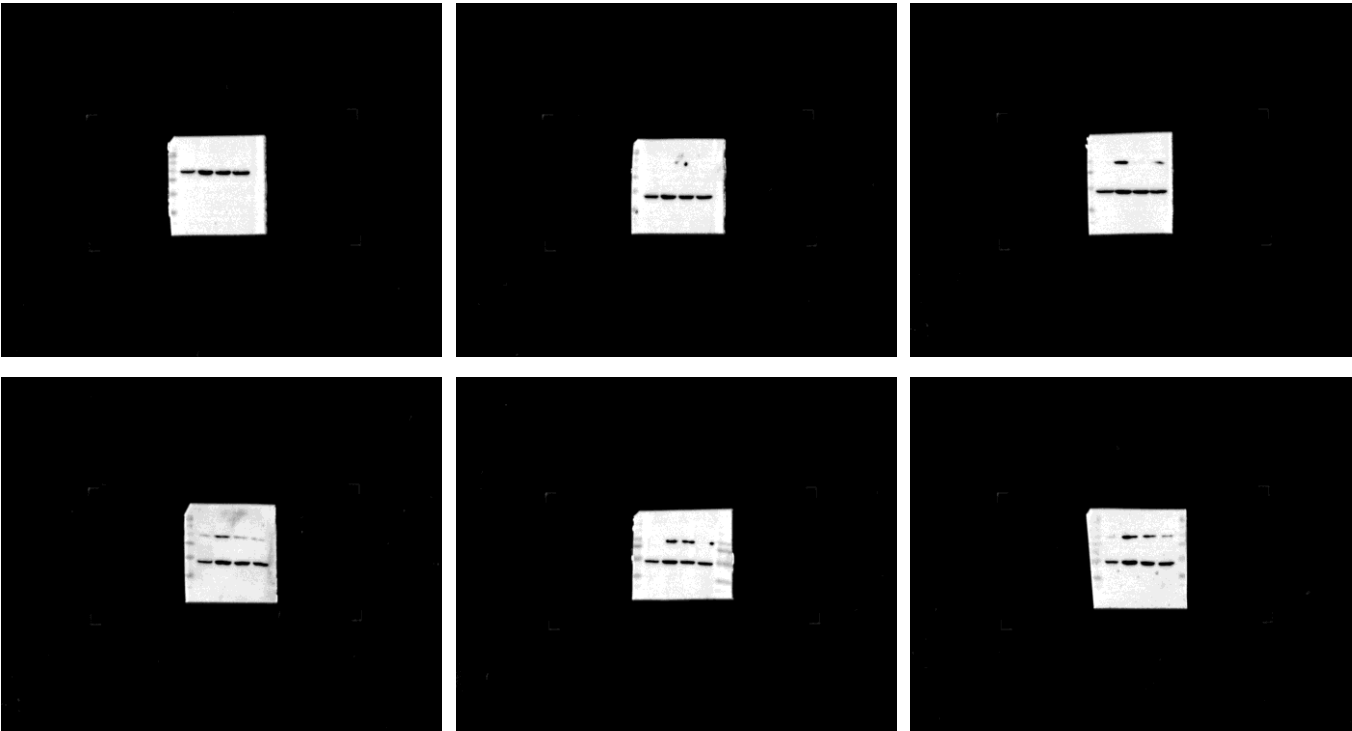

Bcl-2 (n=6)

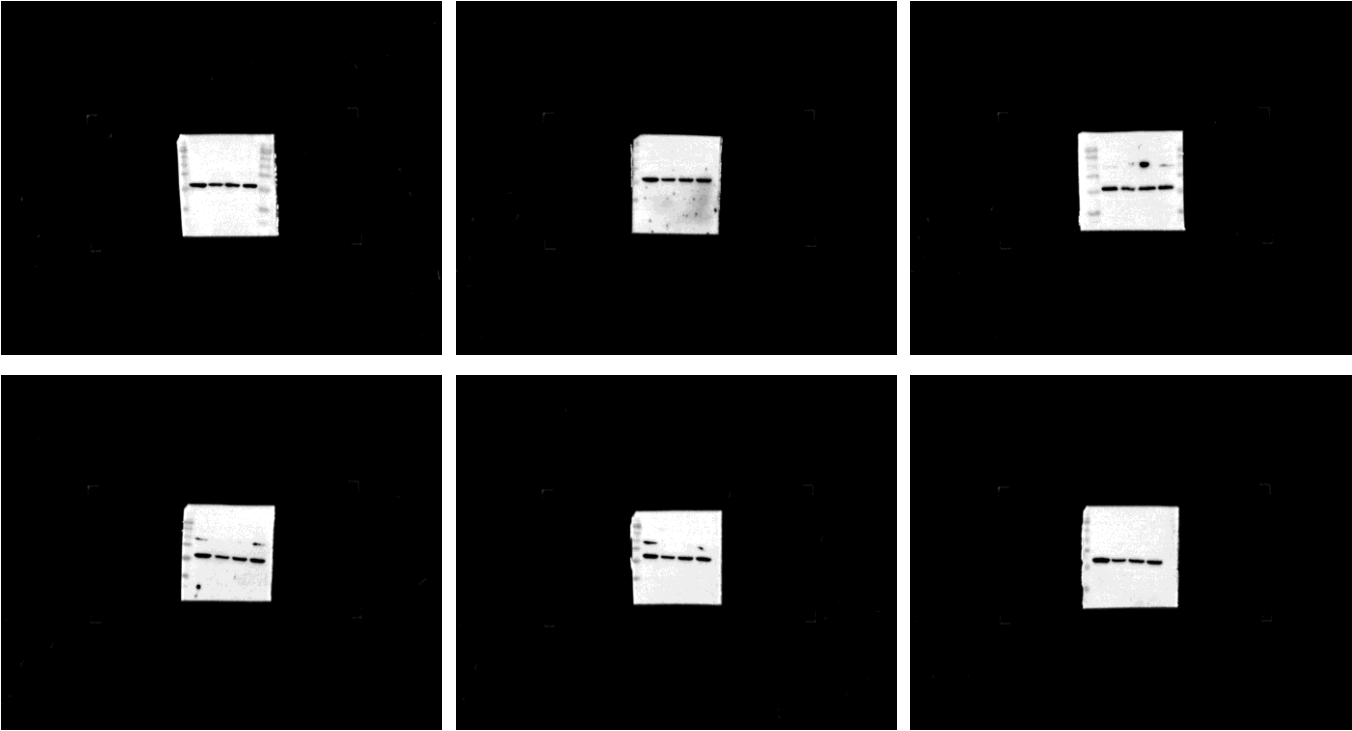

Cleaved-caspase3 (n=6)

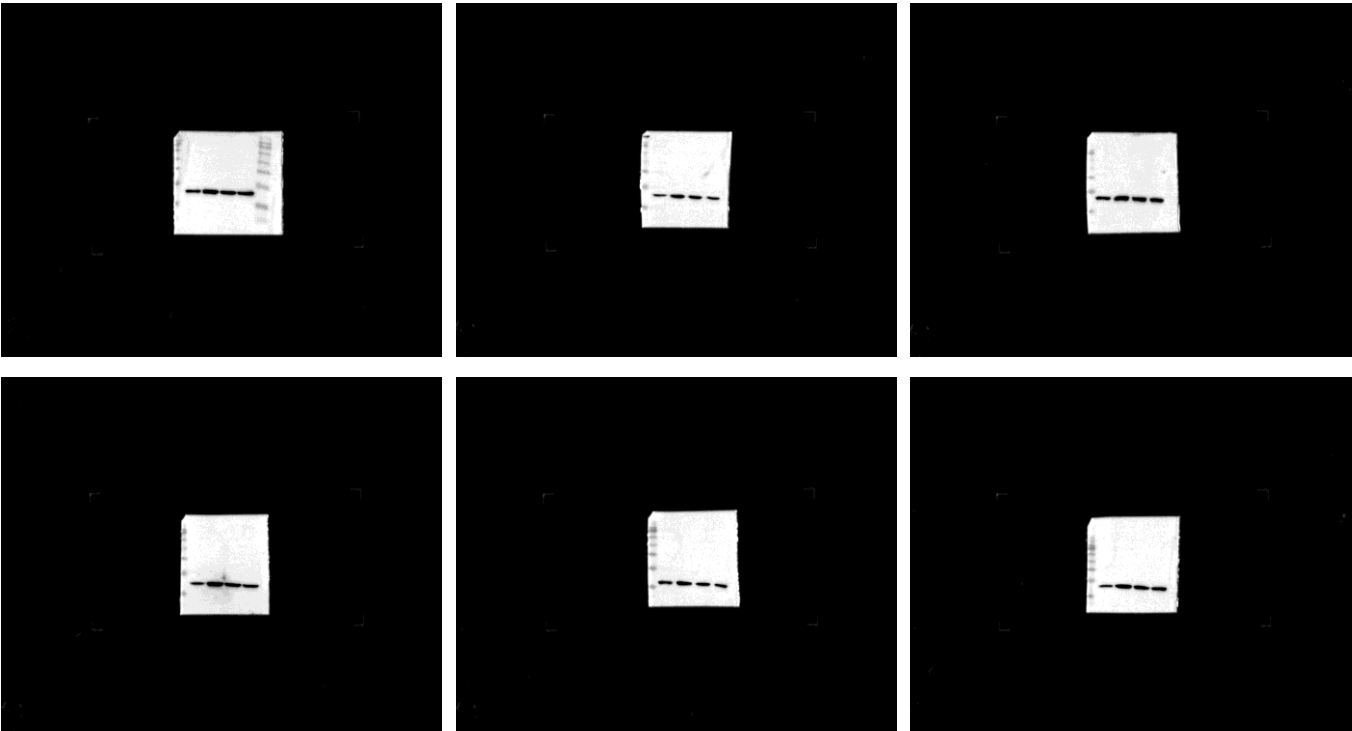

p-PI3K (n=6)

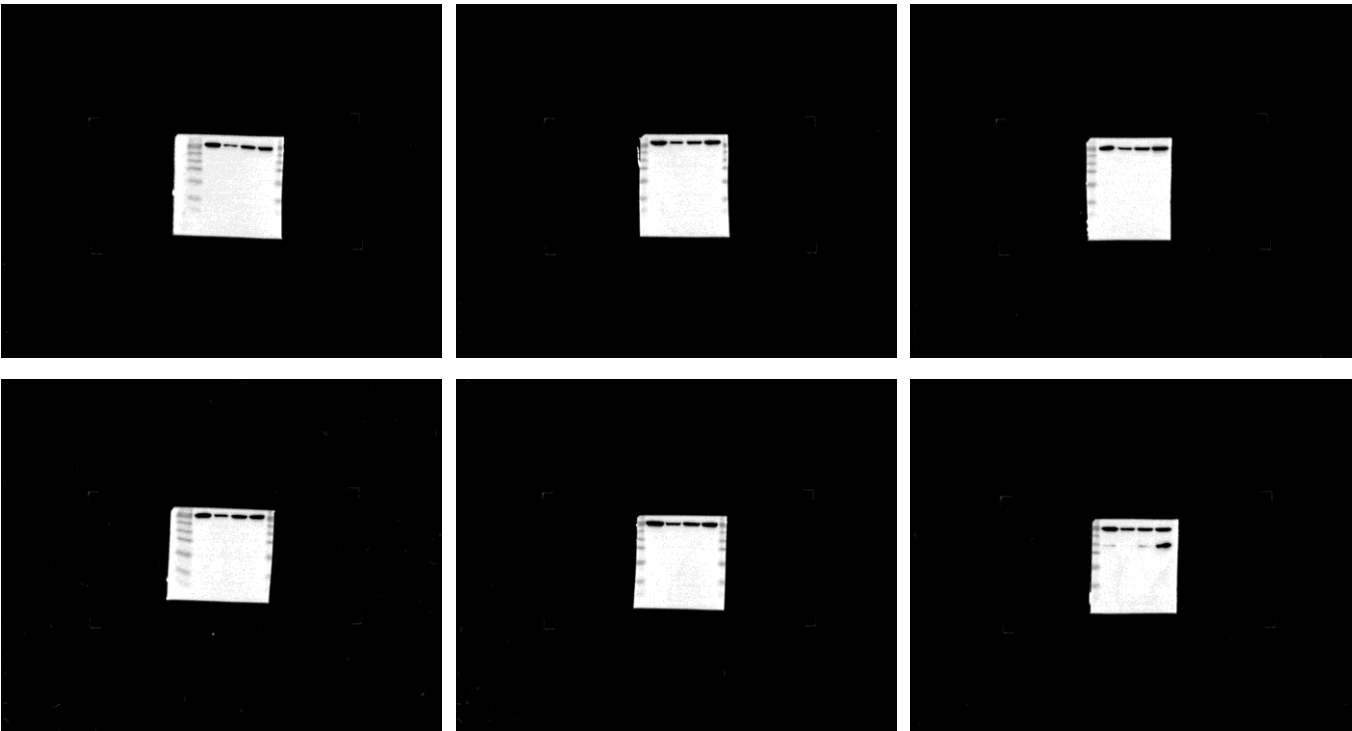

PI3K (n=6)

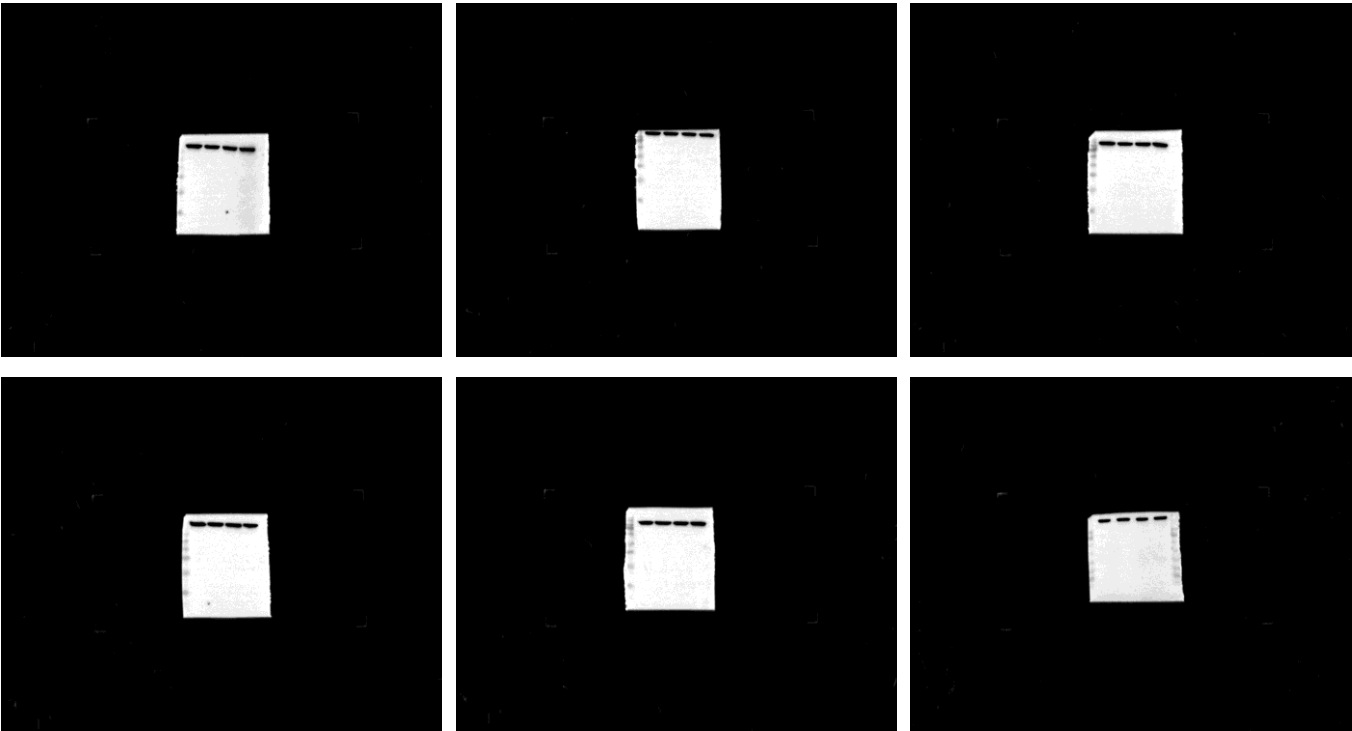

p-AKT (n=6)

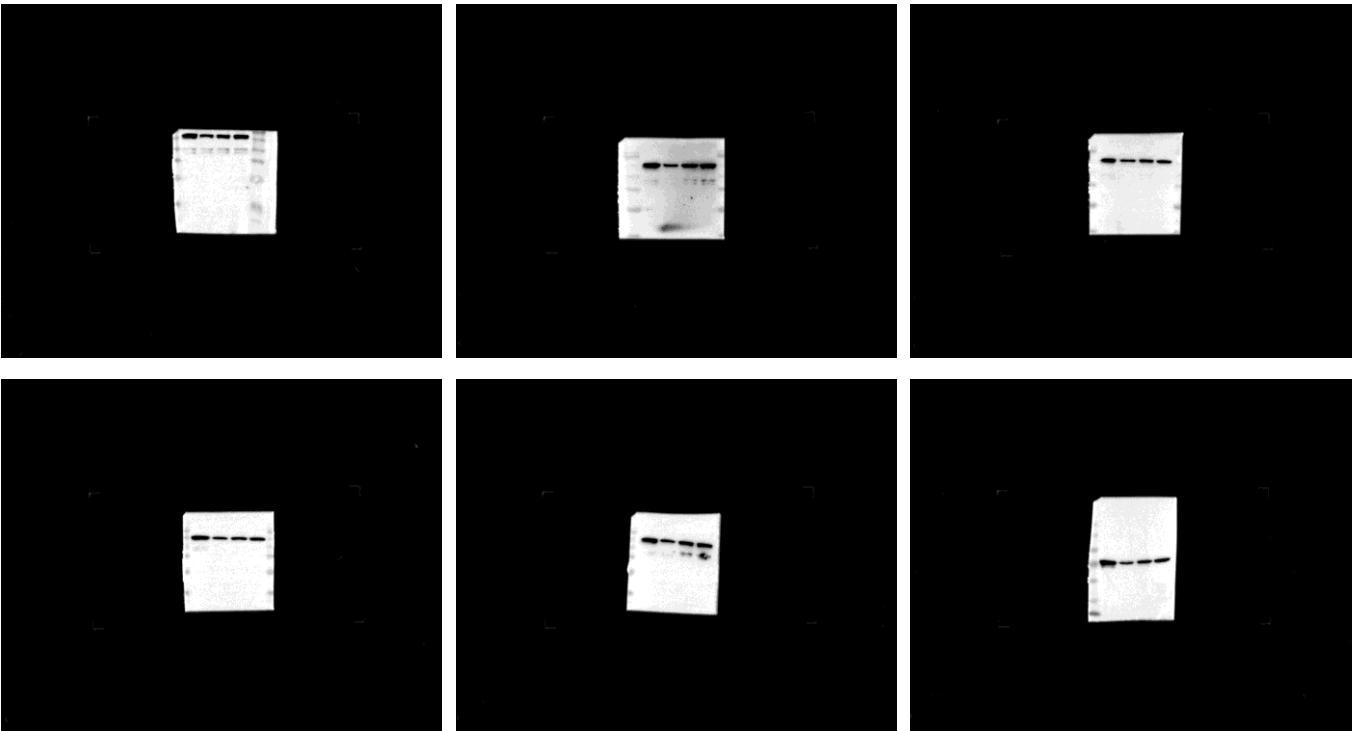

AKT (n=6)

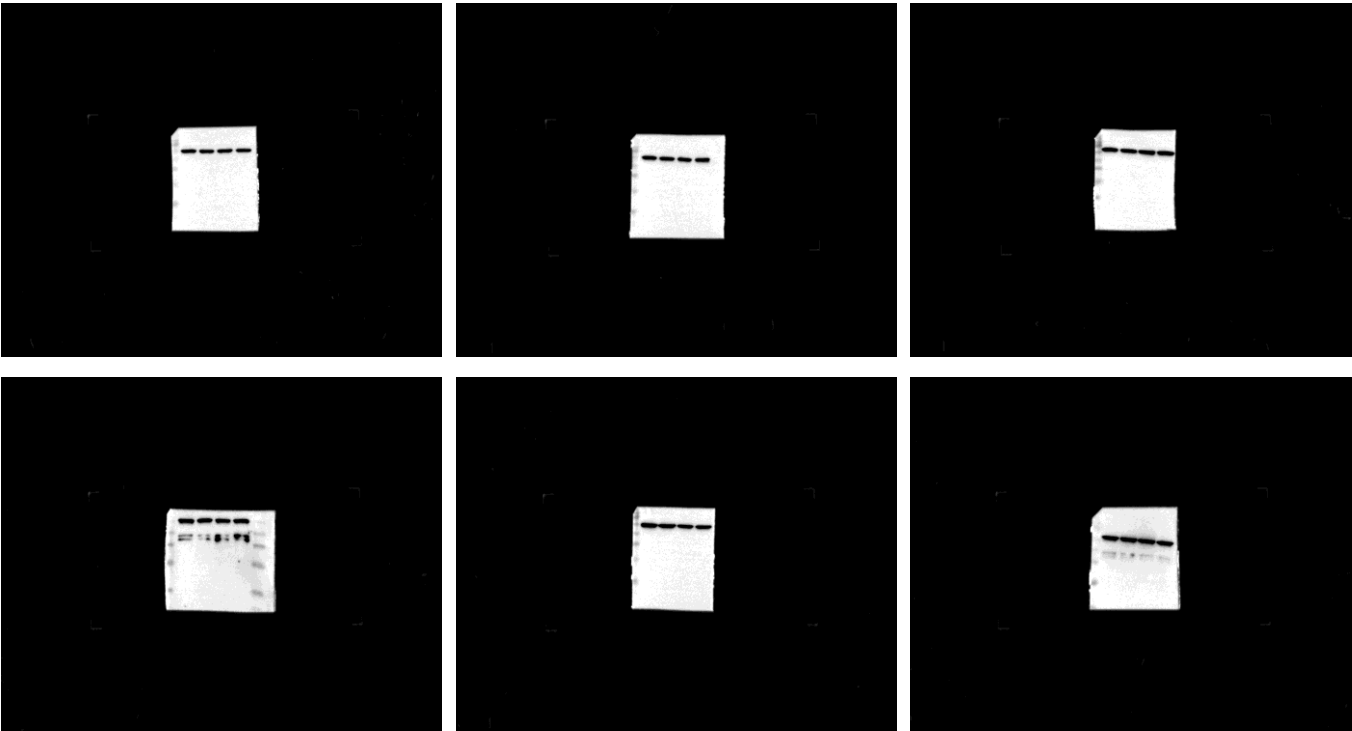

p-Bad (n=6)

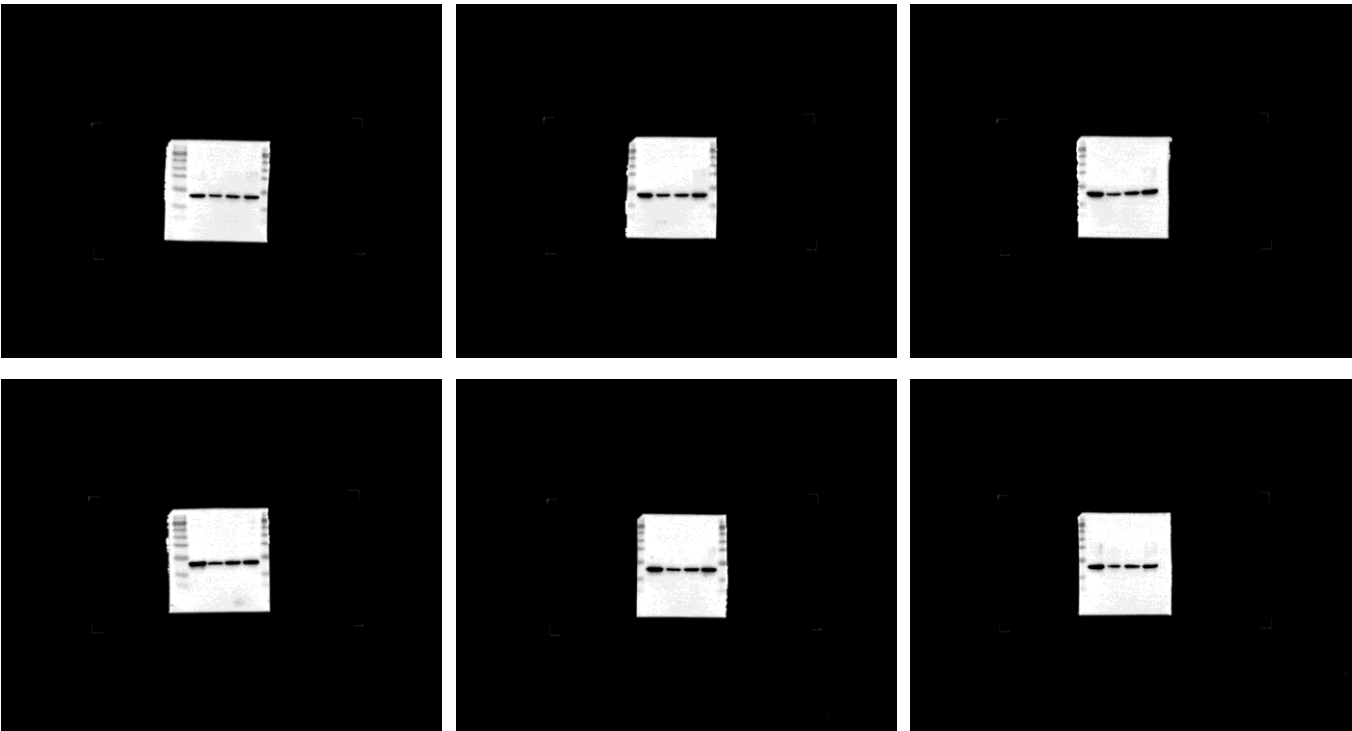

Bad (n=6)

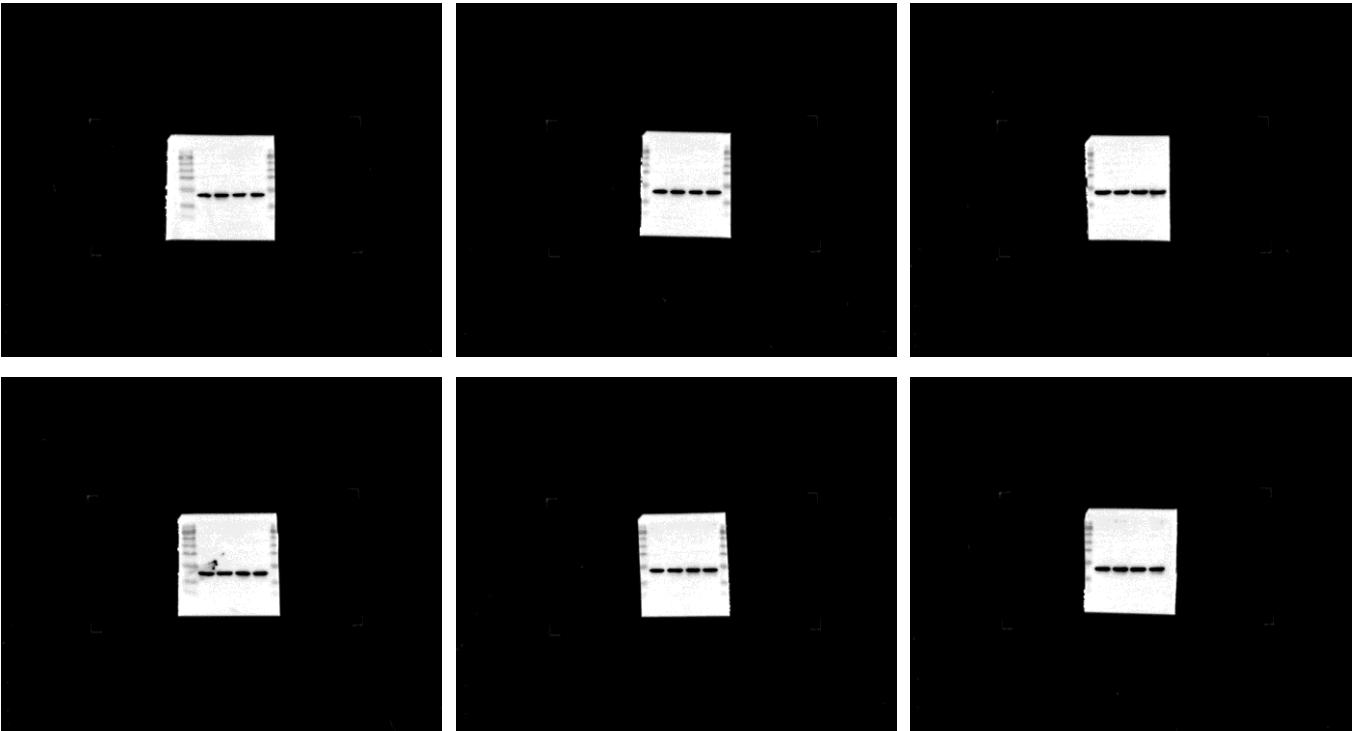

$\beta$ -actin (n=6)

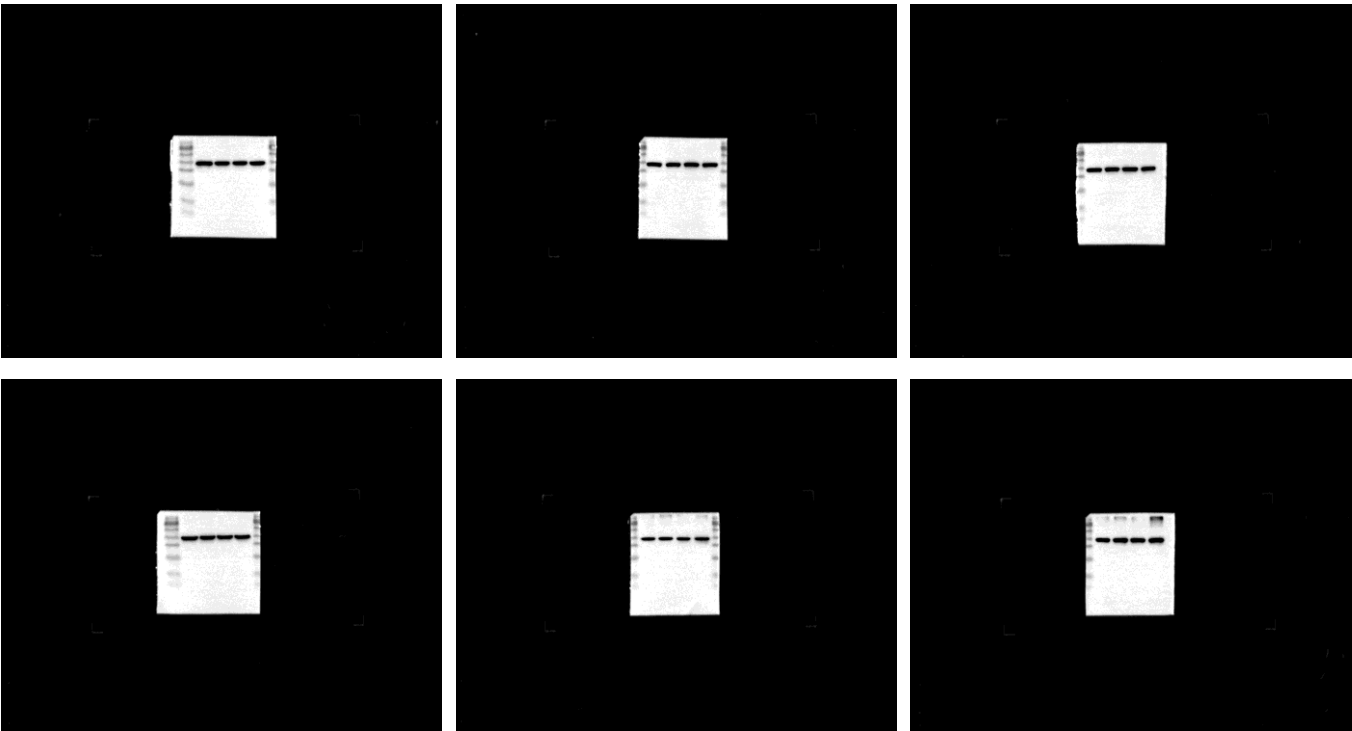

Supplement: S1 Raw images — (PDF) [file pone.0270410.s001.pdf]
